# Phytochemical Profiling and Biological Activities of Pericarps and Seeds Reveal the Controversy on “Enucleation” or “Nucleus-Retaining” of *Cornus officinalis* Fruits

**DOI:** 10.3390/molecules29071473

**Published:** 2024-03-26

**Authors:** Jinyi Zhang, Po Niu, Mingjie Li, Yuan Wang, Yao Ma, Pan Wang

**Affiliations:** 1 School of Mechanical Engineering, Chongqing Three Gorges University, Chongqing 404100, China; zhangjy6100@163.com; 2 Biological Laboratory, HBN Research Institute, Shenzhen Hujia Technology Co., Ltd., Shenzhen 518000, China; limingjie@hbn.cn; 3 School of Agricultural Sciences, Zhengzhou University, Zhengzhou 450001, China; mayao@zzu.edu.cn; 4 Henan Funiu Mountain Biological and Ecological Environment Observatory, Nanyang 474550, China; 5 Sichuan Academy of Agricultural Machinery Science, Chengdu 610066, China; scnjy_wp@163.com

**Keywords:** *Cornus officinalis*, secondary metabolite, α-glucosidase inhibitory activity, cholinesterase inhibitory activity, cytotoxicity

## Abstract

The fruits of *Cornus officinalis* are used not only as a popular health food to tonify the liver and kidney, but also as staple materials to treat dementia and other age-related diseases. The pharmacological function of *C. officinalis* fruits with or without seeds is controversial for treating some symptoms in a few herbal prescriptions. However, the related metabolite and pharmacological information between its pericarps and seeds are largely deficient. Here, comparative metabolomics analysis between *C. officinalis* pericarps and seeds were conducted using an ultra-performance liquid chromatography-electrospray ionization-tandem mass spectrometry, and therapeutic effects were also evaluated using several in vitro bioactivity arrays (antioxidant activity, α-glucosidase and cholinesterase inhibitory activities, and cell inhibitory properties). A total of 499 secondary metabolites were identified. Thereinto, 77 metabolites were determined as key differential metabolites between *C. officinalis* pericarps and seeds, and the flavonoid biosynthesis pathway was identified as the most significantly different pathway. Further, 47 metabolites were determined as potential bioactive constituents. In summary, *C. officinalis* seeds, which demonstrated higher contents in total phenolics, stronger in vitro antioxidant activities, better α-glucosidase and butyrylcholinesterase inhibitory activities, and stronger anticancer activities, exhibited considerable potential for food and health fields. This work provided insight into the metabolites and bioactivities of *C. officinalis* pericarps and seeds, contributing to their precise development and utilization.

## 1. Introduction

*Cornus officinalis* Sieb. et Zucc. is an important medicinal and edible homologous species listed in several versions of the Chinese Pharmacopoeia [1]. The fruits of *C. officinalis* (COFs), consisting of the pericarps (COPs) and seeds (COSs), have been staple materials to treat dementia and some other age-related diseases [2,3,4]. The COPs are mainly used as ingredients for medicinal preparations, while COSs have also been shown to demonstrate some biological activities [5,6]. The pharmacological function of this material is controversial in some herbal prescriptions, which the enucleation (pure pericarps without seeds) and nucleus-retaining (whole fruits, pericarps combined with seeds) have shown similar therapeutic effects for some symptoms. Phytochemical and pharmacological investigations have revealed differences in active constituents and bioactivities between COPs and COSs, but research has mainly focused on a single tissue (either COPs or COSs) [6,7], explaining inaccurate differences in research results due to various factors such as sampling, treatment, test methods, and even the cultivated environment. [8,9,10], and especially the lack of research on the constituents and bioactivities of COSs. Therefore, conducting a comparative investigation of the metabolites and bioactivities of COPs and COSs is of great significance for the precise use of this material.

The active ingredients of plant materials are crucial when they are applied in product processing [11], and they are mainly classified into polysaccharides, flavonoids, phenolics, and alkaloids, each with various biological activities [12,13]. The therapeutic and healthy benefits of COFs are primarily due to the presence of various active ingredients, such as polysaccharides, iridoid glycosides, triterpenoids, organic acids, and saponins, either as a single component or in combination with each other [14,15,16,17,18]. Among these specialized ingredients, iridoids (morroniside, loganin, and cornuside) and triterpenoids (ursolic acid and oleanolic acid) are considered to be the most important components with health-promoting functions [15,16]. Widely targeted metabolomics technology, coupled with various chemometrics, has successfully highlighted the key differential metabolites of test materials [19,20]. In addition, a large number of in vitro assays have been developed to evaluate the bioactivities of the compounds [21,22]. To some extent, reliable detection technology and in vitro assays could help to highlight the differences between COPs and COSs.

In order to clarify the controversy on “enucleation” or “nucleus-retaining” of COFs, the differences in metabolites and bioactivities between COPs and COSs were investigated. Secondary metabolites were quantitatively and qualitatively detected using ultra-performance liquid chromatography-electrospray ionization-tandem mass spectrometry (UPLC-ESI-MS/MS), and several in vitro assays (antioxidant activities, enzyme inhibitory properties, and cytotoxicity test) were evaluated to reveal bioactive differences between COPs and COSs. In addition, the health-promoting constituents retrieved from the traditional Chinese medicine systems pharmacology database were examined to reveal the difference in pharmacology between COPs and COSs. Our results provide a comprehensive comparison that highlights the differences in metabolites and biological activities between COPs and COSs. In particular, the study provides a valuable database for the precise application of both COPs and COSs in the production of food and pharmaceuticals.

## 2. Results and Discussion

### 2.1. Phytochemical Profiling of C. officinalis Fruits

#### 2.1.1. Total Flavonoid, Phenolic, and Alkaloid Content

**Figure 1:**
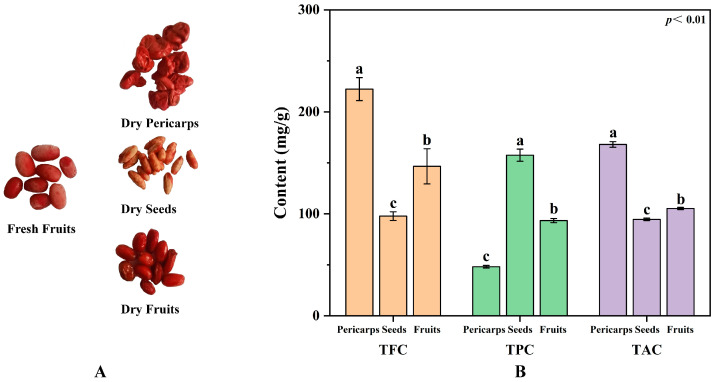
Feature (**A**) and phytochemical profiling (**B**) of *Cornus officinalis* fruits. TFC, total flavonoid content; TPC, total phenolic content; TAC, total alkaloid content; different letters (a, b, and c) above the column diagram indicate significant difference among three groups (*p* < 0.01) for the indicator.

The total flavonoid, phenolic, and alkaloid contents in the extracts obtained were significantly different (Figure 1). The higher total flavonoid content (TFC, 222.31 mg/g) and the alkaloid content (TAC, 168.04 mg/g) were found in the COPs, while a higher total phenolic content (TPC, 157.57 mg/g) was found in the COSs. The level of TFC and TPC in the COPs and COSs were higher than these in native Australian herbs and fruits reported [23]. Selected samples were taken from the same tree at the same time and treated with the same method to eliminate the effects of species, sampling time, sample treatment, and assay methods on the metabolites, demonstrating the true phytochemical differences between COPs and COSs. These phytochemical differences were caused by synthesis differences in the type and content of active constituents between COPs and COSs. In addition, higher levels of ellagic acid (14.51–21.58 mg/g) were found in the COSs [24], while the content of ellagic acid in the COPs were ranged from 1.97 to 4.51 mg/g [25]. Thus, it was found that COPs have a greater potential as a raw material for extracting flavonoids and alkaloids, while COSs have a greater potential for extracting phenolics. Additionally, the study explored the differences between COPs and COFs to determine the feasibility of enucleation (COPs without COSs) and nucleus-retaining (COPs with COSs) are reasonable in certain herbal prescriptions. The study found that the TFC and TAC levels were significantly higher in the COPs compared to the COFs. Conversely, the COFs had a higher TPC than the COPs. It is possible that the similarity in effective components between COPs and COSs for the symptom may be the reason for the alternative use of enucleation and nucleus-retaining in some herbal prescriptions. In general, it is important to distinguish between enucleation and nucleus-retaining when treating diseases due to the difference in active constituents between COPs and COSs.

#### 2.1.2. Secondary Metabolite Profiling

**Figure 2:**
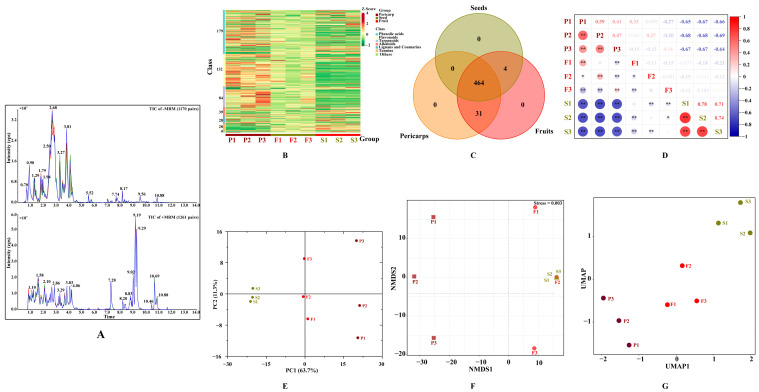
Secondary metabolite profiling of *Cornus officinalis* fruits: (**A**) total ions chromatogram of different quality control samples using multiple reaction monitoring; (**B**) hierarchical cluster analysis; (**C**) Venn diagram; (**D**) correlation analysis; (**E**) principal component analysis; (**F**), non-metric multidimensional scaling; (**G**) uniform manifold approximation and projection. TIC, Total ions chromatogram; +/− MRM, positive and negative multiple reaction monitoring; PC, principal component; P1, P2, and P3 represent samples from pericarps; S1, S2, and S3 represent samples from seeds; F1, F2, and F3 represent samples from fruits; ** in Figure 2D represents significant correlation (*p* < 0.01) and * represents significant correlation (*p* < 0.05); NMDS, non-metric multidimensional scaling; UMAP, uniform manifold approximation and projection.

The secondary metabolites of *C. officinalis* fruits were extracted and extensively detected using UPLC-ESI-MS/MS at both positive and negative ion modes. The high repeatability and stability of metabolite detection can be assessed by comparing the ion chromatogram of three quality control samples (Figure 2A), indicating that the data for test samples were reliable. In all tested samples, a total of 499 secondary metabolites were identified (see Supplementary Excel). These included 179 phenolic acids, 132 flavonoids, 84 terpenoids, and others. The number of metabolites detected in *C. officinalis* fruits was significantly higher than previously identified [15,26], indicating that the use of widely targeted metabolomic technology proved to be an effective method for comprehensively identifying secondary metabolites in this material. The metabolites detected varied across the different samples, with some similarities found within the same tissue (Figure 2B). Among the detected metabolites, 31 were unique to COPs and 4 were unique to COSs (Figure 2C), indicating that secondary metabolites in COPs and COSs were conserved. The unique metabolites may cause the variation in the biological activities between COPs and COSs [23]. A strong correlation was found between the two detected samples based on correlation analysis (Figure 2D). Then, a principal component analysis (PCA) was performed to identify the overall differences and the variations among the samples (Figure 2E). All metabolic variables contributed to a combined variance of 75% in the dataset. The non-metric multidimensional scaling (NMDS, Figure 2F) and uniform manifold approximation and projection (UMAP, Figure 2G) were used to eliminate the quantity on pattern recognition. The study identified two distinct groups associated with COPs and COSs, and grouped together three replicates from the same tissue. The results showed a good repeatability for samples from same tissue and significant differences in overall metabolites between COPs and COSs. The contents and types of bioactive compounds (such as flavonoids, polysaccharide, and morroniside derivatives) were found to be various pharmacological activities in the COFs [2,27]. Enucleation and nucleus retention should be seriously considered for health function due to the difference in active constituents between COPs and COSs.

**Figure 3:**
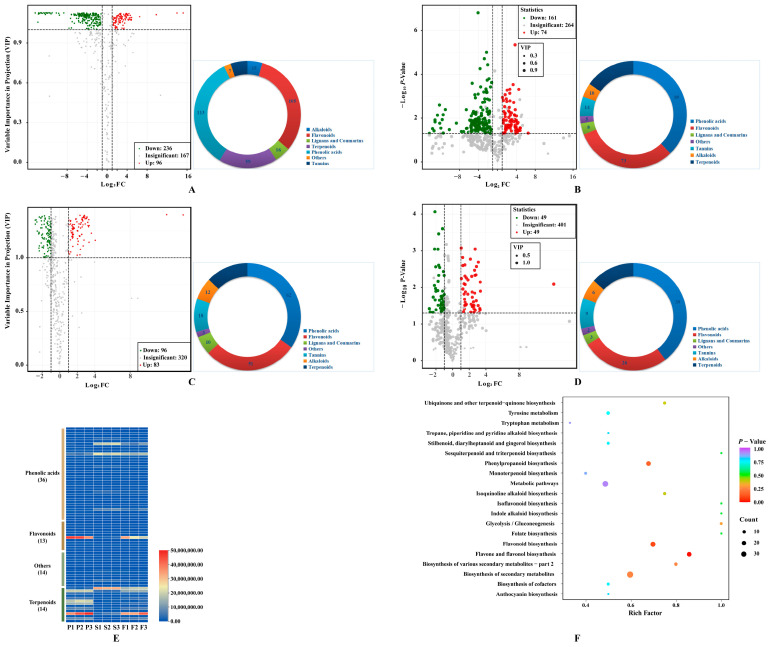
Differentially metabolites: (**A**) Volcano plot of differential metabolites between pericarps and seeds with fold change; (**B**) Volcano plot of differential metabolites between pericarps and seeds with fold change and *p* value; (**C**) Volcano plot of differential metabolites between pericarps and fruit with fold change; (**D**) Volcano plot of differential metabolites between pericarps and fruit with fold change and *p* value; (**E**) key differential metabolites between pericarps and seeds with fold change, *p* value, and false discovery rate; (**F**) enrichment analysis of KEGG pathways of differential metabolites between pericarps and seeds. Pie chart depicts the categories of the differential metabolites; P1, P2, and P3 represent samples from pericarps; S1, S2, and S3 represent samples from seeds; F1, F2, and F3 represent samples from fruits.

Firstly, 332 metabolites were identified in COPs compared to COSs, with 236 downregulated and 96 upregulated metabolites (Figure 3A). The metabolites were classified into seven different classes, with the majority being phenolic acids (113) and flavonols (105). Afterwards, 161 downregulated and 74 upregulated metabolites were filtrated based on a *p* value of less than 0.05 using a *t*-test (Figure 3B). The study identified 235 differential metabolites, which can be classified into 7 categories. The majority of these metabolites were phenolic acids (89) and flavonols (73). Additionally, this study explored the differences in secondary metabolites between COPs and COFs, and identified 179 differential metabolites (96 downregulated metabolites and 83 upregulated metabolites) (Figure 3C). The metabolites can be classified into seven different classes, with the majority being phenolic acids (62) and flavonols (51). Afterward, 49 downregulated and 49 upregulated metabolites were further screened, considering the *p*-value (*p* < 0.05) using a *t*-test (Figure 3D). The study identified 98 differential metabolites, which can be classified into 7 categories. The majority of these metabolites were phenolic acids (39) and flavonols (26). The results indicated that there are differences in secondary metabolites between COPs and COFs, mainly caused by the metabolite differences between COPs and COSs. Finally, the study determined 77 key differential metabolites (51 downregulated and 26 upregulated) in COPs compared with COSs, with a false discovery rate of less than 0.05 (Figure 3E). The pharmacological activities of COPs and COSs may differ due to key differential metabolites. Furthermore, some characteristic metabolites in *C. officinalis* fruits were quantitatively identified (Table S1).

The metabolic pathways of COPs and COSs were compared through KEGG pathway enrichment analysis. The differential metabolites were found to be associated with ‘metabolism’, specifically the metabolic pathway and biosynthesis of secondary metabolites (Figure 3F). The identified differential metabolites were further validated using the KEGG metabolic pathway. The synthesis of these substances was controlled by the flavones and flavonols biosynthesis pathway, phenylpropanoid biosynthetic pathway, and flavonoid biosynthetic pathway. The flavones and flavonols biosynthesis pathway contained 12 metabolites, includingquercetin-3-O-sophoroside, quercetin-3-O-rutinoside, 3,7-di-O-methylquercetin, kaempferol-3-O-glucoside, luteolin-7-O-glucuronide, quercetin-3-O-glucoside, quercetin, quercetin-3-O-sambubioside, kaempferol-3-O-galactoside, kaempferol, kaempferol-3-O-rutinoside, and luteolin-7-O-neohesperidoside (Figure S1). Thirteen metabolites were synthesized in the phenylpropanoid biosynthesis pathway, including 1-O-sinapoyl-D-glucose, *p*-coumaric acid, isoeugenol, chlorogenic acid, coniferin, coumarin, *p*-coumaraldehyde, scopoletin-7-O-glucoside, ferulic acid, sinapinaldehyde, caffeic acid, coniferyl alcohol, and 5-O-p-coumaroylquinic acid (Figure S2). The study identified 16 metabolites in the flavonoid biosynthesis pathway, including dihydromyricetin, eriodictyol, afzelechin, 5-O-p-coumaroylquinic acid, hesperetin-7-O-glucoside, epiafzelechin, naringenin chalcone, kaempferol, aromadendrin, naringenin, chlorogenic acid, quercetin, phloretin-2’-O-glucoside, pinobanksin, isosalipurposide, and naringenin-7-O-glucoside (Figure S3). The flavonoid biosynthesis pathway was found to be the most significantly different pathway between COPs and COSs.

Currently, *C. officinalis* was considered as an excellent functional plant, but the key active constituents with health-promoting functions have not been designated. We queried the identified metabolites in the traditional Chinese medicine systems pharmacology database to identify the key health-promoting constituents. Out of 499 identified metabolites, 47 ones were found to be the chemical compositions of traditional Chinese medicines. These included 15 phenolic acids, 14 flavonoids, 8 lignans and coumarins, 7 terpenoids, 1 tannin, 1 alkaloid, and 1 other (Table S2). The health-promoting constituents identified in *C. officinalis* fruits are associated with treating cancer, tumors, cardiovascular diseases, inflammation, diabetes, Alzheimer’s disease. The study found that many flavonoids and phenolic acids in the *C. officinalis* fruits contribute to these health-promoting functions for humans.

### 2.2. In Vitro Bioactivities of Cornus officinalis Fruits

#### 2.2.1. In Vitro Antioxidant Activities of Extracts

**Figure 4:**
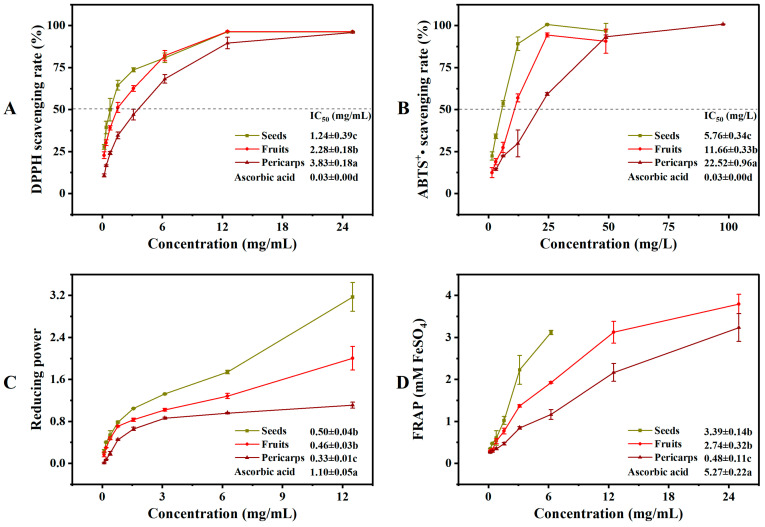
In vitro antioxidant activities of extracts from *Cornus officinalis* fruits, pericarps, and seeds: (**A**) DPPH radical scavenging activity; (**B**) ABTS^+•^ radical scavenging activity; (**C**) reducing power; (**D**) Ferrous reducing antioxidant power. Different letters (a, b, and c) above the column diagram indicate significant difference among three groups (*p* < 0.01) for the indicator; IC_50_, the half maximal inhibitory concentration; DPPH, 2,2-diphenyl-1-picrylhydrazyl; ABTS^+•^, diammonium 2,2′-azino-bis (3-ethylbenzothiazoline-6-sulfonate); FRAP, Ferrous reducing antioxidant power.

The *Cornus* fruits are the most promising natural source of antioxidants [7]. However, there is limited reporting on the difference in antioxidant activity between COPs and COSs, particularly for the antioxidant activity of COSs. A comprehensive antioxidant assay should reflect both free radical scavenging activity and reducing power [22]. The reducing power and ferric ion reducing antioxidant power are typically used to evaluate the reducing capacity of a substance. Higher values indicate a higher antioxidant capacity [28]. Therefore, the antioxidant activities of COFs, COPs, and COSs were tested based on their 2,2-diphenyl-1-picrylhydrazyl (DPPH)and diammonium 2,2′-azino-bis (3-ethylbenzothiazoline-6-sulfonate) (ABTS^+•^) scavenging activity, as well as their reductive power and ferric ion reducing antioxidant power (see Figure 4). The antioxidant activities of solvents extracted from COFs, COPs, and COSs varied significantly. The extracts of COSs showed better antioxidant capacity in terms of DPPH and ABTS^+•^ scavenging activity, as well as reductive power and ferric ion reducing antioxidant power. The variation in antioxidant capacity results may be attributed to the different types and quantities of metabolites in plant extracts [29,30,31]. Previous studies have reported a positive correlation between phenolic components and antioxidant effects [22,32]. Our results confirm this agreement, as the phenolic components and antioxidant activity where the extracts of COSs showed a higher total phenolic content and therefore a higher antioxidant activity compared to COPs. This suggests that COSs could be regarded as a potential antioxidant material for various antioxidant assays.

#### 2.2.2. Enzyme Inhibition Assay of Extracts

**Figure 5:**
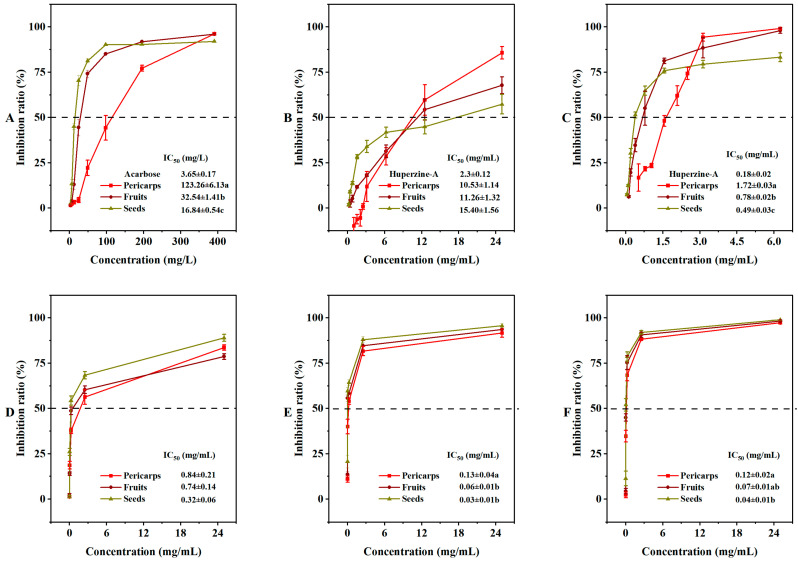
In vitro enzyme inhibition activities and cytotoxicity of *Cornus officinalis* fruits: (**A**) α-glucosidase inhibition activity; (**B**) acetylcholinesterase inhibition activity; (**C**) butyrylcholinesterase inhibition activity; (**D**) cytotoxicity against RAW264.7; (**E**) cytotoxicity against HepG2; (**F**) cytotoxicity against TE-1. IC_50_, the half maximal inhibitory concentration. Different letters (a, b, ab, and c) after the value indicate significant difference among three groups (*p* < 0.01) for the indicator.

Certain traditional medicinal materials have shown significant α-glucosidase inhibitory activity, which can help regulate high blood glucose levels [33,34]. In our extracted samples, the α-glucosidase inhibitory rate decreased gradually in a concentration-dependent manner. The α-glucosidase inhibitory activity of COSs with a lower IC_50_ (16.84 ± 0.54 mg/L) was significantly better than that of COPs (123.26 ± 6.13 mg/L) (Figure 5A). The ability of the extracts to inhibit acetylcholinesterase (AChE) and butyrylcholinesterase (BChE) was also examined (Figure 5B,C). In the AChE inhibition assay, the activity ranking was huperzine-A (2.3 mg/mL) > COP extract (10.53 mg/mL) > COF extract (11.26 mg/mL) > COS extract (15.40 mg/mL). In contrast, the BChE inhibition assay showed a ranking of huperzine-A (0.18 mg/mL) > COS extract (0.49 mg/mL) > COF extract (0.78 mg/mL) > COP extract (1.72 mg/mL). Polyphenols have been reported as effective enzyme inhibitors [35,36]. Our enzyme inhibition assays confirmed the agreement between the phenolic components and enzyme inhibitory activity. The extracts of COSs showed a higher total phenolic content.

#### 2.2.3. Cytotoxicity of Extracts

The RAW264.7 cells were often used to evaluate the cytotoxicity of various extracts or active ingredients [37]. The inhibitory effects of COFs, COPs, and COSs against RAW264.7, HepG2, and TE-1 cells were in a dose-dependent manner (Figure S4). The extracts of COFs, COPs, and COSs exhibited stronger cytotoxicity against HepG2 and TE-1 cells than RAW264.7 cells, suggesting that they could be regarded as potential anticancer agents against HepG2 and TE-1. The extracts showed significant differences in inhibitory effects against HepG2 and TE-1 cells, but not against RAW264.7 cells (Figure 5D–F). Previous studies have reported a positive correlation between phenolic components and anticancer effects [38], which is consistent with our findings that the extracts of COSs with higher total phenolic content exhibited greater anticancer activity. Therefore, COSs that exhibit a lower IC_50_ against HepG2 and TE-1 cells compared to COPs could be considered as a potential anticancer material with regard to the cell inhibitory assays.

## 3. Materials and Methods

### 3.1. Sample Collection and Preparation

Fresh COFs were obtained from a plantation at Xixia County of Henan Province, China (famous home of the species, 111°46′7.94″ E, 33°37′25.91″ N, 850 m). Composite COFs were randomly collected from a healthy and productive tree when the fruits can be dropped easily. Sixty fruits were chosen for similarities in color and size. Thirty COPs and COSs were separated from thirty COFs. The COFs, COPs, and COSs were freeze dried using a YTLG-10A vacuum freeze drier (Shanghai Yeto Technology Co., Ltd., Shanghai, China) and then crushed to a homogeneous powder.

### 3.2. Sample Extraction

In total, 200 mg of frozen powdered samples and 5 mL of 60% methanol (*v*/*v*) were mixed into a centrifuge tube. The solutions were ultrasonic for 30 min at 30 °C in a XM-5200UVF ultrasound bath (Xiaomei Ultrasonic Instrument Co., Ltd., Suzhou, China) and then centrifuged at 12,000 rpm for 10 min at 4 °C (Eppendorf Centrifuge 5424R, Eppendorf China Ltd., Shanghai, China). The supernatant was collected, and the residue was extracted and centrifuged twice. A total of nine supernatant liquids with three biological replicates for each sample were obtained and filtrated with quantitative filter papers. The obtained solutions were set to 15 mL; 5 mL of the solutions were used for metabolomic analysis, and the others were evaporated using a reduced-pressure rotary evaporator (Yarong Biochemical Instrument Factory, Shanghai, China) and freeze-dried.

### 3.3. Phytochemistry Profiling of Cornus officinalis Fruits

#### 3.3.1. Determination of Total Flavonoids, Phenolics, and Alkaloids

The study detected the TFC, TPC, and TAC of the samples using spectrophotometric methods, as described in previous research [13]. The TFC was expressed as rutin equivalent (RE) per gram of dry sample weight (mg RE/g DW) using the aluminum chloride colorimetric method at 510 nm. TPC was calculated as gallic acid equivalent (GAE) per gram of dry sample weight (mg GAE/g DW) using the Folin–Ciocalteu assay at 765 nm. TAC was estimated as chelerythrine equivalent (CE) per gram of dry sample weight (mg CE/g DW) using the acid dye colorimetric method at 470 nm with a TECAN SPARK multimode reader (Männedorf, Switzerland).

#### 3.3.2. Identify and Quantify Determination of Secondary Metabolites

The extracted solution was filtered through a 0.22 μm filter membrane before being analyzed using UPLC-ESI-MS/MS analysis (UPLC, SHIMADZU NexeraX2; MS, Applied Biosystems 4500 Q TRAP, Shanghai, China). The analytical conditions were as follows: injection volume of 4 μL, temperature of 40 °C, Agilent SB-C18 column (1.8 µm, 2.1 mm × 100 mm), and a mobile phase consisting of solvent A (pure water with 0.1% formic acid, chromatographic grade with purity of more than 98%) and solvent B (acetonitrile with 0.1% formic acid) with a linear gradient and a flow rate of 0.35 mL/min. The gradient elution was performed using solvent A. The solvent composition was changed as follows: 0–11.0 min 95%-5% A, 11.0–12.0 min 5%-5% A, 12.0–12.1 min 5%-95% A, and 12.1–15.0 min 95%-95% A. The ESI parameters were set as follows: temperature, 550 °C; turbo spray voltage, 5500 V/−4500 V; ion source gas I, gas II, and curtain gas, 50, 60, and 25.0 psi, respectively. Instrument tuning and mass calibration were performed using 10 and 100 μmol/L polypropylene glycol solutions in triple-series quadrupole mass spectrometry and linear ion trap modes, respectively.

For the qualitative analysis of metabolites, the primary and secondary MS data were used to annotate metabolites based on the Metware database (MWDB) (Wuhan Metware Biotechnology Co., Ltd., Wuhan, China) and the public metabolite database.

#### 3.3.3. Determination and Annotation of Differential Metabolites

The study screened differential metabolites by comparing the fold change of metabolites between two samples, where the value was ≥2 (upregulated) or ≤0.5 (downregulated) in COPs compared with COSs or COFs. The variable importance in projection value of the orthogonal partial least squares-discriminant analysis (OPLS-DA) was also considered, with a threshold of not less than 1. Key differential metabolites were determined, taking into account a false discovery rate of less than 0.05 [39]. The metabolites were identified and annotated using the Kyoto Encyclopedia of Genes and Genomes (KEGG) database.

#### 3.3.4. Determination of the Key Health-Promoting Constituents

The detected metabolites were searched in the traditional Chinese medicine systems pharmacology database. The key health-promoting constituents were determined when oral bioavailability was more than 5% and drug-likeness was more than 0.14 [40], and related-disease information of determined constituents was also obtained.

### 3.4. Determination of In Vitro Bioactivities of Cornus officinalis Fruits

#### 3.4.1. Determination of In Vitro Antioxidant Assays

The antioxidant assays were evaluated using 2,2-diphenyl-1-picrylhydrazyl (DPPH) and diammonium 2,2′-azino-bis (3-ethylbenzothiazoline-6-sulfonate) (ABTS^+•^) scavenging activities together with ferrous-reducing antioxidant power (FRAP) and reducing power (RP), as described in previous research [13]. For these in vitro antioxidant assays, the same volume of methanol was used as a blank control, and ascorbic acid was used as a positive control.

#### 3.4.2. Enzyme Inhibition Assay

The study evaluated the level of cholinesterase inhibition using AChE) and BChE, as described in previous research [13], and α-glucosidase inhibition assay was conducted at 405 nm, as described in a reported method [27]. The same volume of phosphate-buffered saline (PBS) was used as a blank control, while huperzine was used as a positive control for cholinesterase inhibition assay, and acarbose was used as a positive control for α-glucosidase inhibition assay.

#### 3.4.3. Determination of Cytotoxicity

The DAPI staining assays were executed to evaluate the cytotoxicity against RAW 264.7, HepG2, and TE-1 cells [41]. All cells were in the logarithmic growth phase (2.5 × 10^5^ cell/mL), and the number of living cells was more than 95%. The cells were incubated in a 12-well plate for 24 h, and different extracts were added. The mixtures were cultured for 24 h, and then 2.5 μg/mL of DAPI (100 μL) was added. The plate was washed three times using PBS after removing DAPI stain. An AMG Evos M5000 Inverted Fluorescence Microscope (Westover Scientific, Inc., Seattle, DC, USA) was used to photograph, and Image J FIJI software (National Institutes of Health, Bethesda, MD, USA) was used to count the cells.

#### 3.4.4. Determination of the Half Maximal Inhibitory Concentration

The scavenging rates (%) of free radicals (DPPH and ABTS^+•^), inhibiting rates (%) of enzymes (AChE, BChE, and α-glucosidase) and cytotoxicities against RAW 264.7, HepG2, and TE-1 cells were calculated as (blank–sample)/blank × 100. The half maximal inhibitory concentration (IC_50_) is the mass concentration of the sample at a 50% inhibition, the lower IC_50_ values indicate the higher inhibitory capacity [13]. The curve was plotted according to the inhibitory rates of the samples at different concentrations (25.00 mg/mL of initial concentration, decuple dilution method for cytotoxicity and twofold half dilution method for others).

### 3.5. Statistical Analyses

One-way analysis of variance and Pearson’s correlation tests (*p* < 0.01) were conducted on IBM SPSS 19.0 statistical software (SPSS Inc., Armonk, NY, USA). The filtered metabolite data was submitted to R 4.1.3 (www.r-project.org, accessed on 29 December 2021, Auckland, New Zealand) for hierarchical cluster analysis and OPLS-DA (Access date: 29 December 2021). Column diagram, line charts, PCA, Venn diagram, correlation analysis, NMDS, and UMAP were conducted using Origin 2021 software (Originlab, Northampton, MA, USA).

## 4. Conclusions

Systematically and comprehensive studies demonstrated similarities and dissimilarities of metabolites and in vitro bioactivities between COPs and COSs. In this study, a total of 499 secondary metabolites (179 phenolic acids, 132 flavonoids, 84 terpenoids, and others) were detected in *C. officinalis* fruits (pericarps and seeds) based on widely targeted metabolomic technology. Among them, 77 metabolites were determined as key differential metabolites between COPs and COSs, and the flavonoid biosynthesis pathway was identified as the most significantly different pathway between COPs and COSs. Further, 47 metabolites were determined as potential bioactive constituents. In conclusion, COSs showed higher contents of TPC, stronger in vitro antioxidant activities, better α-glucosidase and BChE inhibitory activities, and stronger anticancer activities, exhibiting considerable potential for food and health fields. This work provided first-hand comprehensive information on the metabolites with health-promoting functions for humans, and also provided insight into the metabolites and bioactivities of COPs and COSs.

## Data Availability

Data are contained within the article or Supplementary Materials.

## Supplementary Materials

The following supporting information can be downloaded at: https://www.mdpi.com/article/10.3390/molecules29071473/s1. Supplementary Excel Secondary metabolites identified in *Cornus officinalis* fruits. Figure S1 Differential metabolites in flavones and flavonols biosynthesis pathway. Figure S2 Differential metabolites in phenylpropanoid biosynthetic pathway. Figure S3 Differential metabolites in flavonoid biosynthetic pathway. Figure S4 Inhibitory effects against RAW264.7, HepG2, and TE-1 cells. Table S1 Quantitative determination of characteristic metabolites in C. officinalis fruits. Table S2 Key active compounds in Cornus officinalis fruits queried in the traditional Chinese medicine systems pharmacology database.
