# Supplementary material for: Phytochemical Profiling and Biological Activities of Pericarps and Seeds Reveal the Controversy on “Enucleation” or “Nucleus-Retaining” of Cornus officinalis Fruits
Source: Molecules. 2024 Mar 26;29(7):1473. doi: 10.3390/molecules29071473 (PMC11012811; doi:10.3390/molecules29071473)
Supplement: Supplementary file 1 [file molecules-29-01473-s001.zip › Supplementary materials.pdf]

Supplementary Materials

Figure S1 Differential metabolites in flavones and flavonols biosynthesis pathway

Figure S2 Differential metabolites in phenylpropanoid biosynthetic pathway

Figure S3 Differential metabolites in flavonoid biosynthetic pathway

Figure S4 Inhibitory effects against RAW264.7, HepG2, and TE-1 cells

Table S1 Quantitative determination of characteristic metabolites in *C. officinalis* fruits

Table S2 Key active compounds in *Cornus officinalis* fruits queried in the traditional Chinese medicine systems pharmacology database

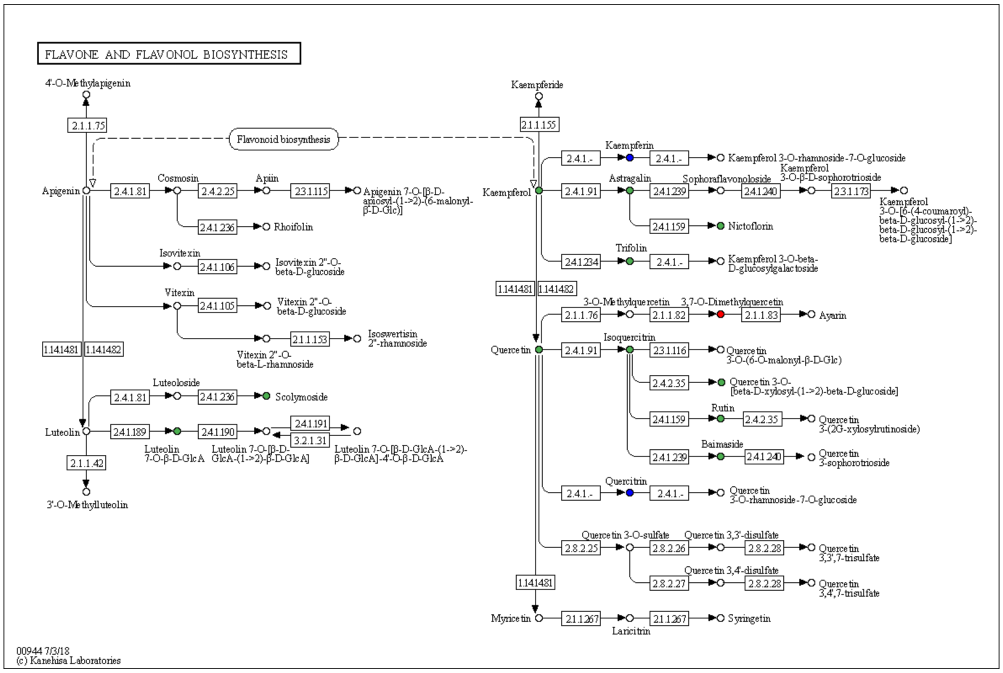

Figure S1. Differential metabolites in flavones and flavonols biosynthesis pathway

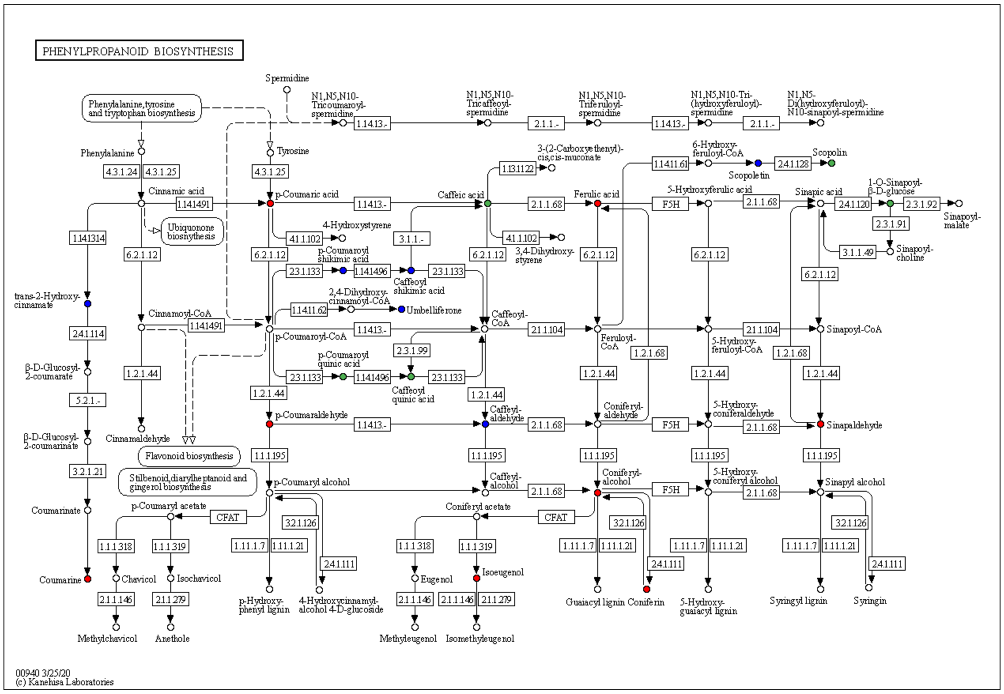

Figure S2. Differential metabolites in phenylpropanoid biosynthetic pathway

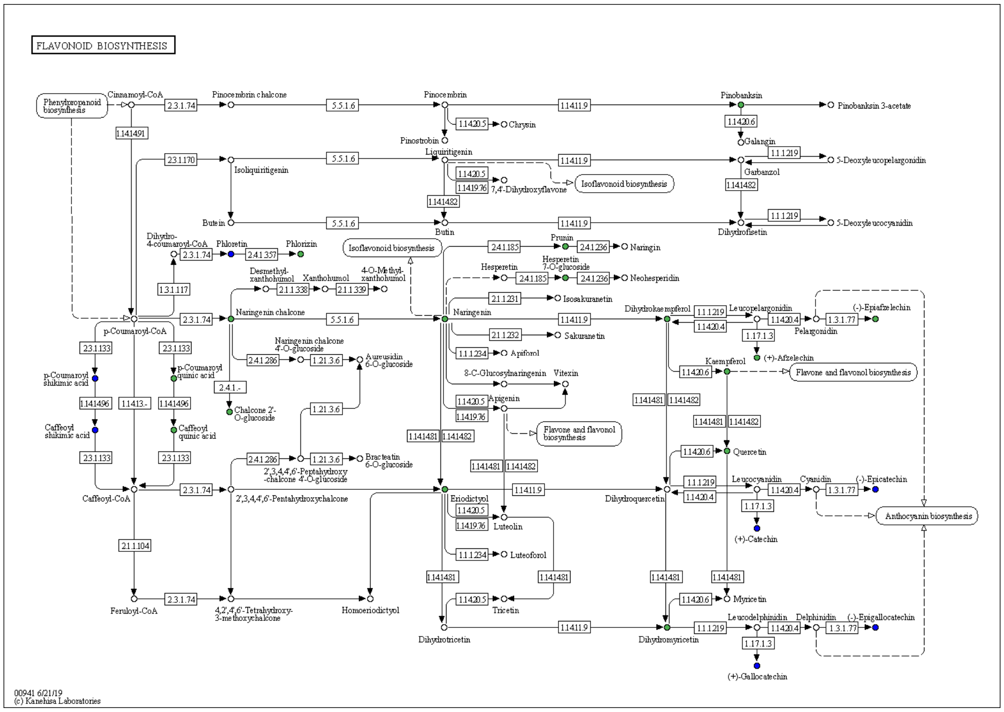

Figure S3. Differential metabolites in flavonoid biosynthetic pathway

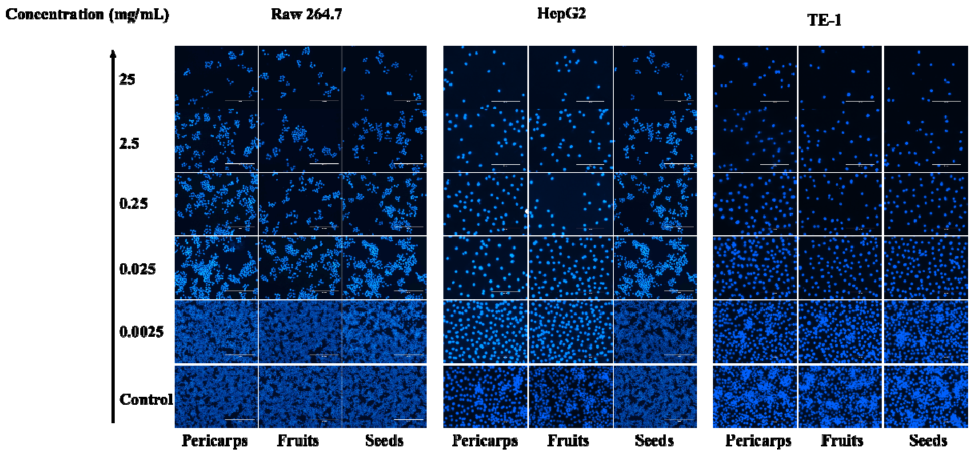

Figure S4. Inhibitory effects against RAW264.7, HepG2, and TE-1 cells

Table S1. Quantitative determination of characteristic metabolites in *C. officinalis* fruits

Compounds	CAS	Fruit	Pericarp	Seed
3,4-Dihydroxy-5-methoxybenzoic acid	3934-84-7	12.70±1.30	16.35±0.44	3.74±0.17
β arbutin	497-76-7	21.18±0.52	18.71±2.40	12.43±0.36
(-)-catechin	18829-70-4	25.08±0.46	27.54±1.12	22.61±0.84
Cornin	548-37-8	7.44±1.52	0.79±0.26	16.92±0.56
Cornuside	131189-57-6	1.78±0.01	1.84±0.16	0.13±0.01
Gallic acid	149-91-7	0.19±0.01	0.05±0.01	0.40±0.01
Gynesine	535-83-1	0.23±0.00	0.39±0.07	0.07±0.00
Loganin	18524-94-2	10.86±0.14	11.71±0.29	10.41±0.30
Morroniside	25406-64-8	3.52±0.10	1.93±0.12	10.60±0.32
Sweroside	14215-86-2	10.80±0.26	1.15±0.13	25.88±0.63

Table S2. Key active compounds in *Cornus officinalis* fruits queried in the traditional Chinese medicine systems pharmacology database

**Compounds**	**Class**
3-hydroxybenzaldehyde	Phenolic acids
4-Hydroxyacetophenone	Phenolic acids
Protocatechualdehyde	Phenolic acids
2-Methoxy-4-methylphenol	Phenolic acids
Isovanillin	Phenolic acids
4-MethoxycinnaMaldehyde	Phenolic acids
p-Coumaric acid	Phenolic acids
Ethylparaben	Phenolic acids
Homogentisic acid	Phenolic acids
Salicin	Phenolic acids
Digallic Acid	Phenolic acids
Androsin	Phenolic acids
Diisooctyl Phthalate	Phenolic acids
4-Hydroxybenzoic acid	Phenolic acids
Isoeugenol	Phenolic acids
7-O-Methyleriodictyol	Flavonoids
Dihydrokaempferide	Flavonoids
Diosmetin	Flavonoids
Tangeretin	Flavonoids
Nobiletin	Flavonoids
Kaempferol	Flavonoids
Quercetin	Flavonoids
Azaleatin	Flavonoids
Patuletin	Flavonoids
Kaempferol-7-O-rhamnoside	Flavonoids
Quercetin-3-O-arabinoside	Flavonoids
Kaempferol-3-O-glucoside	Flavonoids
Isohyperoside	Flavonoids
Myricetin-3-O-rhamnoside	Flavonoids
Coumarin	Lignans and Coumarins
Umbelliferone	Lignans and Coumarins
Fraxetin	Lignans and Coumarins
Scopoletin-7-O-glucoside	Lignans and Coumarins
Dehydrodiisoeugenol	Lignans and Coumarins
Lariciresinol	Lignans and Coumarins
Veraguensin	Lignans and Coumarins
Syringaresinol	Lignans and Coumarins
Geniposidic acid	Terpenoids
Geniposide	Terpenoids

Asperulosidic acid	Terpenoids
Oleanolic acid	Terpenoids
Betulinic acid	Terpenoids
Ursolic acid	Terpenoids
Tormentic acid	Terpenoids
Betaine	Alkaloids
Chebulic acid	Tannins
5-Hydroxymethylfurfural	Others
